# Supplementary material for: Revisiting the Loa loa microfilaremia thresholds above which serious adverse events may occur with ivermectin treatment
Source: PLoS Negl Trop Dis. 2025 Mar 28;19(3):e0012957. doi: 10.1371/journal.pntd.0012957 (PMC11981195; doi:10.1371/journal.pntd.0012957)
Supplement: S1 Table — (DOCX) [file pntd.0012957.s001.docx]

**Supporting information to: Revisiting the *Loa loa* microfilaremia thresholds above which serious adverse events may occur with ivermectin treatment. Boullé *et al*.**

**Table S1. Multivariable logistic regression coefficients for probabilities of post-IVM SAEs according to the *L. loa* MFD and individual characteristics**

|  | Coefficient | p-value | 95% Confidence Interval |
| --- | --- | --- | --- |
| $Loa_{FP1}$ | 2.563691 | <0.001 | [1.950504 - 3.176879] |
| Age category | |  |  |
| [<20] (reference) |  |  |  |
| [21-30] | 1.102808 | 0.219 | [-.6558337 - 2.861449] |
| [31-40] | 1.966523 | 0.027 | [.2247915 - 3.708255] |
| [41-50] | .6945951 | 0.462 | [-1.155194 - 2.544384] |
| [>50] | .4595063 | 0.613 | [-1.321245 - 2.240257] |
| Male sex | 1.049903 | 0.020 | [.1666049 - 1.933201] |
| Constant | -11.55906 | <0.001 | [-14.14612 - -8.971998] |
