# Supplementary material for: Revisiting the Loa loa microfilaremia thresholds above which serious adverse events may occur with ivermectin treatment
Source: PLoS Negl Trop Dis. 2025 Mar 28;19(3):e0012957. doi: 10.1371/journal.pntd.0012957 (PMC11981195; doi:10.1371/journal.pntd.0012957)
Supplement: Attachment — Submitted filename: Response_to_reviewers_auresp_2.docx [file pntd.0012957.s004.docx]

**Reviewers' comments:**

**Summary and General Comments**

Reviewer #1: I’d like to thank the authors for the careful consideration of all the comments that were made the reviews. I feel that the manuscript has strongly improved.

We appreciate the reviewer’s insightful comments and their consideration of our responses.

However some comments remain:

-Line 85/86: please do add a remark that the association does not necessarily imply causality

Response: Done “, although causality has not yet been established” (line 86)

-Line 129: explain abbreviation q.d.

Response: Done, “*quaque die* [q.d., each day]” (line 130)

-Introduction, last paragraph: although various treatments for loiasis are being introduced in the introduction, the paper is only about ivermectin. This should be mentioned in the last paragraph of the introduction. Also, some rationale should be provided as to whý this choice is made.

Response: Done, “This study focuses on IVM which is often used before, or preferred to DEC due to its availability – when DEC is frequently unavailable – and efficacy in reducing MFD.” (lines 144-145)

-Line 201: incomplete sentence

Response: We apologize, the sentence was indeed meant to be linked with the following sentence. This was corrected: “To model the occurrence of an SAE, we (…)” (now line 205)

-Line 215: add explanation on how the various multivariable models are compared. (Based on AIC? Or deviance?)

Reponse: Done “using the AIC” was added after “were compared” line 218. The acronym was introduced after Akaike Information Criterion “(AIC)” in line 215.

-Line 223: add the term “multivariable” to clarify that you switch to discussing the multivariable models here (before it was about bivariate models)

Response: Done (line 217)

-S1 Table: clarify in the table legend that these coefficients are for the multivariable model.
Response: Done, “multivariable” was added before “logistic regression coefficients” in the table tile.

-Lines 281-291: only the coefficients for males aged 31-40 are significantly different from 0 (see Table S1). However, the text also emphasizes differences for 21-30 year olds and for 41-50 year olds. I am not sure whether that is justifiable. This should be corrected or, at a minimum, discussed.
Response: If it is true that only coefficient for males aged 31-40 is significant in multivariable analysis, the text lines 281-291 and the corresponding table (Table 3) present results of estimates accounting for age, but also for male sex (also significant, see Table S1) and Loa MFD (idem), therefore it depends on each combination of sex, MFD, and age, that are each pictured in one cell of the associated Table 3. The statistical significancy of each predicted SAE probability (lower bound of 95%CI >0) was pictured in Table 3 (*, see table legend), which is the case for all the above-mentioned categories except for Males aged 41-50. Nevertheless, we aimed to estimate the MFD threshold at which the predicted risk exceeds 2.5% (and not 0), and this is what we have indicated in the text without being able to associate a p-value (that would be for >0 and not >2.5) with it.

-Lines 295-311: Please clarify at the start of this paragraph what point you are addressing. I guess this relates to the comment from reviewer 3 that your risk estimates are way higher than expected from the number of SAEs reported to MDP? It is now very hard for the reader to understand why you are bringing all this information up.

Response: That is very true, and we thank the reviewer for their careful attention to clarity for readers after our modifications. “Our risk estimates are overall higher than expected from the number of SAEs reported to MDP” was added at the start of the paragraph. (lines 303-304).

-Line 397: replace “extrapolation” by “generalization”

Response: Done

-Lines 425-426: do we have any idea what proportion of (individually treated) patients have such high mf counts? Will this change have important implications for practice? Also, do you recommend that this lowered threshold for ivermectin treatment is also applied in test and not treat programmes for onchocerciasis? It may be worthwhile to clarify this also in the conclusion.

Response: An ongoing French study, yet unfinished and unpublished estimates that 7.8% of treated patients over the 2000–2022 period had MFD>8000 mf/ml, but it is not yet finalized and therefore cannot be mentioned yet. This threshold is applicable when thick blood smears are drawn for individual treatment but regarding T&NT programmes for onchocerciasis it is difficult to provide a clear answer, that would require a new study and analysis to further investigate the correlations and variability between the loascope and the thick blood smear. This was added to the discussion “To simplify therapeutic recommendations, the threshold of 8000 mf/mL could thus be recommended for all individually treated patients (i.e. with TBS available). Further studies should investigate the correlation and variability between the LoaScope and the TBS to guide programmatic recommendations.” (lines 334-337)
