# Supplementary material for: Revisiting the Loa loa microfilaremia thresholds above which serious adverse events may occur with ivermectin treatment
Source: PLoS Negl Trop Dis. 2025 Mar 28;19(3):e0012957. doi: 10.1371/journal.pntd.0012957 (PMC11981195; doi:10.1371/journal.pntd.0012957)
Supplement: Attachment — Submitted filename: Response to reviewers.docx [file pntd.0012957.s003.docx]

**Reviewers' Comments:**

**Objectives**

Reviewer #1: Objectives:
The study concerns a re-analysis of previously collected data. The analysis is very similar to the one published previously by the same group (Chesnais et al, ref 49 in the current paper). This earlier analysis is not cited in the introduction and the rationale for redoing the analysis with only marginally different methods is not explained. The main difference between the current and previous analysis relates to how the analysis is framed (related to the treatment of Loa loa infection or related to SAE prevented by not treating Loa loa cases in MDA programmes for onchocerciasis). The authors now choose for a logistical model that includes age, while previously they preferred a logistical model that did not include age, although models including age were also presented in ref 49! I think the authors should explain better in the introduction why their earlier analyses are not sufficient for the purpose described in the current paper (and hence why the current paper is worth publishing) and how they are improving on the earlier analysis.
Response O1:

We thank the reviewer for highlighting the need to better explain the rationale for reanalyzing previously published data and the improvements introduced in the current analysis.

To address this, we have revised the introduction to explicitly outline the purpose and added value of the current study compared to the earlier analysis by Chesnais *et al*.

While the earlier study focused primarily on identifying the association between *L. loa* MFD and severe adverse events (SAEs) in the context of MDA programs for onchocerciasis, therefore destined to a public of specialists with a focus on modelling studies, the present analysis aims to provide a more nuanced and practical understanding of SAE risks for clinical management purposes, destined to non-specialist physician (general infectious diseases practitioner) whose practice relies more on pre-established nomograms or thresholds rather than mathematical equations. In conclusion, the main interest of this paper is that it is not aimed at the same audiences.

Specifically, the current study introduces two key improvements compared to the analysis by Chesnais et al:

1. Inclusion of age in the logistic regression model: While the earlier analysis explored models with and without age, but with age as a second-order fractional polynomial (FP2) transformation, this study systematically integrates age categories as a covariate to account for its confounding effect, thereby enhancing the robustness of the risk predictions.

2. Refined focus on clinical implications: The current analysis estimates SAE probabilities for specific MFD cutoffs, stratified by age and sex, to guide treatment decisions extending beyond the scope of MDA programs.

These improvements address gaps in the earlier analysis and underscore the importance of revisiting the data to provide actionable insights for individual patient management.

The last paragraph of the introduction was therefore modified to:

“While previous analyses [Ref Chesnais et al., 2017] modeled the relationship between *L. loa* MFD and SAEs in the context of MDA programs, the current study focuses on refining risk stratification to guide clinical management. This analysis systematically includes age category as a covariate to account for potential confounders and provides detailed SAE probability estimates stratified by MFD cutoffs, age, and sex. The objective of this paper is to operationalize the results of previous modeling, to provide turnkey information to prescribers on the risks incurred for a given MFD, sex and age, and to provide additional elements for reflection on these risks.”

Statistical methods: The authors state how loa MFD was included in their previous logistic models (line 195-196), but not how they do it in the current paper (e.g. line 197 and 204-206). I assume that they are still included is as a continuous variable after FP1 transformation. Did they use the exact same transformation as before (and which transformation was that?) or did they retest all candidate transformations again? This should be stated explicitly.

Response O2: The *L. loa* MFD was indeed included with the same FP1 transformation, but we agree that our wording was not sufficiently explicit in the previous version of the manuscript.

This was clarified to answer this and ‘Minor comments’ #4 & #5 in the ‘Summary and General Comments’ from the same reviewer. The beginning of the statistical analyses paragraph was modified as follows: “We constructed bivariate models using age, sex, and pre-treatment *L. loa* MFD as covariables. A first-order fractional polynomial (FP1) transformation of the *L. loa* MFD was used [49].

Age was incorporated as a categorical variable (≤20, 21-30, 31-40, 41-50, >50). This transformation was guided by the shape of the second-order FP polynomial (FP2) relationship of age with the occurrence of SAEs [49].

The bivariate model using this categorical categorization had minimal difference in Akaike Information Criterion when compared to the FP2 transformation and was chosen to avoid the use of an equation for age and to provide more easily readable estimates for end-users.”

Also, the authors should clarify how they derived the SAE probabilities presented in tables 2 and 3. Did they use the logistic equations to calculate the risk at the given cutoffs for table 2 and table 3? Similarly, how did they estimate the safety threshold presented in the last paragraph of the results section? Were they simply red from the graphs in the supplementary information?

Response O3: A sentence was added to the methods section “Margins post-estimation commands in Stata 17.0 were used to obtain predicted probabilities after estimating odds ratios in logistic regression.”

Reviewer #2: The methods are clear and appropriate.

Reviewer #3: The objectives, design, sample size, statistical analysis are appropriate.

**Results**

 Reviewer #1: The presentation of results is somewhat confusing, as table 2 seems to be based on their previously reported analysis (ref 49). Table 3 and figure 1 are new, but parameters of the logistic regression equations are not provided. Please provide the equations with information on confidence intervals around parameter estimates. Table 3 and figure 1 seem to present the same information, where predicted probabilities are extracted from the graph at given cut-off values for table 3 (not a bad idea, as the figure panels are too small for readers to extract such numbers themselves).

Response:
We added the regression equation in the result section:

”

$P_{SAE}=\frac{1}{1+e^{-(2.563691 \times Loa_{FP1}+1.049903\times Sex +\beta_{n}\times Age_{cat}-11.55906)}}$	(3)

with : $\beta_{n} \left\{ \begin{aligned} \beta_{0}=0 \\ \beta_{1}=1.102808 \\ \beta_{2}=1.966523 \\ \beta_{3}=0.6945951 \\ \beta_{4}=.4595063 \end{aligned} \right.$ for $\left\{ \begin{aligned} Age_{cat_{0}}\left[ <20 \right] \\ Age_{cat_{1}}\left[ 21-30 \right] \\ Age_{cat_{2}}\left[ 31-40 \right] \\ Ag{e_{cat}}_{3}\left[ 41-50 \right] \\ Age_{cat_{4}}\left[ >50 \right] \end{aligned} \right.$

with $Age_{cat}=0$for reference category [<20] and $Age_{cat}=1$ otherwise, $Sex=0$ for females and $Sex=1$ for males. $Loa_{FP1}$ is defined in equation (1) in the Material and methods section.”

Risk probabilities were not extracted from the graphs, although they do represent the same information, but computed using post-estimations commands (margins) in Stata (see response O3).

It is not clear why in table 3 the cells are color-coded, while the color coding is in a separate column in table 2.
Response: We apologize for the confusion introduced, as there was no specific reason for this difference in presentation. Table 2 was modified to match presentation of table 3.

Figure 1: explain the interpretation of the black line and grey area in the caption.

Response: The black line represents post-estimation risk, and the grey area the 95%CI. This was precised in the figure legend. “Predicted probabilities (black line) with their 95% confidence interval (grey area).”

Reviewer #2: The results address the research question and are supported by the figures.

Reviewer #3: Results are adequately presented

**Conclusions**

Reviewer #1: The discussion section is too long and presents a lot of information that is not directly linked to the analyses performed. I recommend to shorten it considerably, maybe even cut down the number of words by one-third or so.

Response: We thank the reviewer for this valuable comment, that aligns with another comment of reviewer 2. Discussion has been shortened by 25% (while integrating new comments).

We recognize that while our intention was to provide a comprehensive overview of the pathophysiology and to integrate state-of-the-art knowledge on other molecules such as Albendazole (ALB) and Diethylcarbamazine (DEC), this approach may have diluted the central message of the manuscript. In response to this valuable feedback, we have significantly reduced the discussion of SAEs related to ALB and mostly DEC. By doing so, we aim to tighten the narrative and ensure that the primary focus remains on the differences in SAE risk related to IVM treatment among Loa loa-infected individuals, particularly with respect to age and sex. The revised discussion now emphasizes the IVM results and their implications, as suggested by the reviewer.

Some limitations of the analysis are described. For me, the main limitation is the lack of data in general: inferences are based on data from only 2 studies with 38 SAE occurrences. There may be a risk of overfitting of the data. Another aspect to address: to what extent are results generalizable to other countries?

Response: We added in the limitation a sentence on the risk of over-fitting of the data, nonetheless those are the only available data to guide treatment administration to date, reason why we believe it is worthy. See “Although there is a risk of over-fitting due to the paucity of data, all available data was used in the present study in order to guide treatment administration.” (lines 445-446)

Regarding the generalizability of the results, while it is true that the data come exclusively from studies conducted in Cameroon, we believe that the findings may be applicable to other areas where *L loa* is endemic. This is because the geographic distribution of loiasis is relatively restricted, and the mechanisms of SAE following ivermectin treatment are likely similar across endemic regions. However, we cannot ascertain the generalizability, and therefore added a statement in the limitations section to reflect this point:

“Indeed the data were collected exclusively in Cameroon, but we assume that the findings are likely generalizable to other endemic regions – or to patients outside endemic areas but who contracted the disease in Central Africa – but differences in clinical practices and on other comorbidities between countries may limit direct extrapolation.”

A statement about the added value of the current analysis over the previously presented one (in ref 49) may be useful.

Response: See response O1.

Reviewer #2: The conclusions section needs substantial work. Right now it does not hold together well. It is trying to cover way too much in the way of case studies for not only IVM but DEC and ALB-associated SAEs. As a result it dilutes the overall message of this paper and the reader quickly becomes confused as to the purpose of the manuscript. The Discussion would be greatly improved if it focused on the impact of IVM on SAEs (with no more than a paragraph each on how the results might differ for DEC and ALB, based on experiences documented to date).

Response: We thank the reviewer for highlighting the need to refine the focus of our discussion (see also same response for reviewer 1, above). We recognize that while our intention was to provide a comprehensive overview of the pathophysiology and to integrate state-of-the-art knowledge on other molecules such as Albendazole (ALB) and Diethylcarbamazine (DEC), this approach may have diluted the central message of the manuscript. In response to this valuable feedback, we have significantly reduced the discussion of SAEs related to ALB and mostly DEC. By doing so, we aim to tighten the narrative and ensure that the primary focus remains on the differences in SAE risk related to IVM treatment among *Loa loa*-infected individuals, particularly with respect to age and sex. The revised discussion now emphasizes the IVM results and their implications, as suggested by the reviewer.

Reviewer #3: The authors indicate that their results indicate that these thresholds for individual treatment guidance correspond to mean risks of SAEs around 0.1% for 8000 mf/mL and around 2.5% for 30,000 mf/mL. However this doesn’t correspond at all with the evidence we have from the Ivermectin donation program where 5 billion tablets have been donated and very little SAE or AE have occurred. This cannot be explained by underreporting. The prevalence of AE and SAE would be much higher if their conclusion was to be true. That should at least be considered in the discussion.

Response:

We thank the reviewer for raising this important point. While it is difficult to provide a precise answer to this observation, we consider several factors that could explain the apparent discrepancy between the SAE risks estimated in our study and the data reported from the MDP:

According to Vinkeles et al., in 1995 (we added the reference also in the manuscript), there were an estimated 122,300 individuals with hypermicrofilaraemia (defined as >20,000 mf/mL) in areas where MDA was implemented. Based on our study’s estimated SAE risk of ~1% at this threshold, approximately 1,200 SAEs could have been expected annually, as compared with the total 500 encephalopathies (not including therefore non-neurological serious adverse events). In addition, it is important to note that after a first IVM treatment, MFD is significantly reduced, likely lowering the risk of subsequent SAEs in these individuals. This has been outlined in the study by Pion et *al.*, who found that among individuals with MFD >20,000 mf/mL, only 1 out of 57 remained above this threshold one year after. This underscores the effect of repeated ivermectin administration in reducing MFD and preventing the development of high MFDs, and the subsequent reduction in SAE risk over time.

Although the MDP has robust reporting mechanisms, it is possible that not all SAEs were systematically recorded, particularly in the earlier phases of the program before the implementation of active surveillance. In conclusion, we believe that although there is a slight discrepancy from a programmatic view, those data remain coherent, but that they are realistic when considering the individual risk.

We agree that this point warrants consideration in the discussion and we therefore modified the beginning of the discussion to reflect these considerations and address the discrepancy raised by the reviewer:

“Vinkeles et al. [53] estimated that in 1995, there were 122,300 individuals with MFD ≥20,000 mf/mL in MDA regions, corresponding to ~1,200 expected SAEs annually based on our estimates that SAE-risk above 20,000 mf/mL is close to 1%. Furthermore, repeated IVM treatments significantly reduce MFD, where only 1 out of 57 individuals remained above the 20,000 mf/mL threshold after one year [16], further diminishing the at-risk population for the next IVM round. In addition, repeated CDTI probably prevent the development of hypermicrofilaremia in individuals who still have MFDs below the risk threshold. While the discrepancy is acceptable from a programmatic perspective, the individual risk estimates remain plausible. The number of SAEs reported to the MDP is probably underestimated given the flaws in the AE surveillance system in some countries, particularly in remote areas where TBS are not always prepared to assess the post-treatment MFD required to characterize “probable” or “possible” cases, and before the implementation of community-directed treatment with IVM (CDTI), that allowed improved case identification and management over time.”.

**Editorial and Data Presentation Modifications?**

Reviewer #1: (No Response)

Reviewer #2: This paper needs careful review by a native English speaker. There are quite a few minor grammatical errors and awkward phrasings. Some of which are shared below:
Line 42: Errant symbol after the “%”

Response: ‰ is the symbol for 1/1000 and not % corresponding to 1/100.
Line 49: Replace “them” with something more specific like, “these high risk groups”

Response: “Them” was initially used because of length restrictions. We changed it by “these high risk groups” and removed “previously used” in next line (line 50) to compensate the word balance.
Line 88: Remove “being”

Response: Corrected
Line 104: Rather than “during”, I suggest “for” or “over the course of”; what does b.i.d. stand for? Response: Response: ‘During’ was replaced by ‘for’. B.i.d. stands for *bis in die* which is a common abbreviation for “twice a day” in the medical Anglo-Saxon literature. The precision was added.
Line 138: The use of “recalls” doesn’t make sense here, unless this is just a personal communication (and if so the individual should be attributed).
Line 257: “bond” should be “bound”

Response: Corrected
Line 280: instead of “have also” should be “also have”

Response: Corrected
Line 281-282: Do you mean a continuum related to MFD? If so, please state explicitly.

Response: We refer to a continuum in the severity of the adverse events, which could therefore be underpinned by the same pathophysiological mechanism. “in the severity” was added to the sentence to clarify it.
Line 289: Missing an “and” before strongyloides.

Response: Corrected
Line 290: consider “shedding light on” instead of “putting the light on”

Response: Corrected
Line 290: should be “In the absence”

Response: Corrected
Line 334: should we “were noted”

Response: Corrected
Line 388: Should be “imbalance”

Response: Corrected
Line 391: “In driven” should be “driven”

Response: Corrected

Reviewer #3: (No Response)

**Summary and General Comments**

Reviewer #1: MINOR COMMENTS:

1) Line 76: does it only hamper elimination programmes for onchocerciasis, or also those for lymphatic filariasis?
Response: The main concern is for elimination programmes for onchocerciasis, reason why we mentioned only onchocerciasis, although it had also affected those for LF earlier.

2) Line 84: a causal relation between loiasis mf density and excess mortality has not been proven. The cited studies merely demonstrate that there is an association, but other factors could explain the association (e.g. spending much time in the forest may not only be associated with loiasis, but may also entail other risks that could lead to mortality; socio-economic status; etc). Rephrase this sentence.
Response: The sentence was modified to “and two studies have shown that it may be associated with excess mortality”

3) Line 142: the paper is not about AEs, but about SAEs. Rephrase the objective accordingly.
Response 3: Indeed, the sentence was rephrased accordingly.

4) Line 195/196: not necessary to say what was done in a previous study. Just explain what you do in the current study.
5) Line 225/226: can this information be moved to the methods section?

Responses 4 & 5: See response O2.

6) Line 226/227: remove "Using the MFD thresholds used in the literature dealing with L. loa related SAEs". Response 6: This part of the sentence was removed

7) Line 308: prognostic ==> prognosis

Response 7: Corrected

8) Line 403: please start a new paragraph for a discussion on the limitations.

Response 8: Corrected

9) Line 416/417: "that surveillance after treatment of hypermicrofilaremic individuals with this drug should be strictly monitored" ==> unclear, consider rephrasing

Response 9: The sentence was rephrased to: “hypermicrofilaremic individuals receiving ALB should be carefully monitored, possibly in a hospital setting”

Reviewer #2: This manuscript has an important message to relay about the differences in SAE risk related to IVM treatment among Loa loa infected individuals, based on age and sex. The title states that it is revisiting the thresholds related to SAEs due to IVM treatment; however, the manuscript spends a lot of time describing SAEs due to Albendazole and DEC. At the moment the main message of the paper is easily lost with all the description of specific cases and specific treatment strategies. I strongly suggest tightening up the paper and focusing more on the IVM results and the implication in the discussion.
Response: This comment was taken into account as part of the response to the ‘Conclusion’ comment of the same reviewer #2.

Some additional more minor revision requests to strengthen the paper:
Lines 249-258: There is not sufficient explanation of how the exact thresholds reported here (e.g. 2,410 mf/ml) were calculated. Are these the values that represent the exact MFD corresponding to the risk of SAEs (eg, 0.005%)? If so, why is it that they are said to approximate the a priori thresholds (e.g. 2,000 mf/ml threshold) and not the other way around. I find this paragraph confusing.

Response: The margins post-estimation command was used to calculate the exact MFD corresponding to the given risk of SAEs. A sentence was added to the discussion to clarify that this was how they were calculated.

We apologize for this confusing paragraph. Table 2 presents calculated risk in univariate logistic analyses corresponding to the usual literature thresholds, for instance 20,000 mf/ml corresponded to 1.01% SAE risk. We then calculated what was the exact value of MFD associated with 1.00% risk so that we could use a cut-off in terms of %, and for instance for 1.00% it was 19,890 mf/mL.

We removed the confusing sentences, and added a small paragraph below Table 2 to clarify, as follows:

“The other way around, using rounded percentages, and calculating the exact corresponding MFD gave the following correspondences: for 0.005% it was 2,410 mf/mL, for 0.1% it was 7,935 mf/mL, for 1% it was 19,890 mf/mL, and for 2.5% it was 28,815 mf/mL.”

Lines 263-267: For these MFD thresholds by age and sex at the 2.5% risk threshold, can you provide a CI around each threshold to give a sense of how stable the value is? I know you can get a rough sense from Figure 1, but it would be helpful to report them in a table.

Response: “As the prediction range, we reported the two MFD values having 1% or 2.5% risk as their lower or higher bound of their 95%CI of predicted risk, respectively.”

Values were added to the paragraph lines 263-267 as requested by the reviewer for the 2.5% risk threshold, and also to the 1% risk threshold that they requested in a later comment:

“For some specific sex and age categories, the rounded threshold to reach a predicted risk >2.5% (corresponding to the predicted overall risk when MFD exceeds 30,000 mf/mL) was lower than 30,000 mf/mL. Specifically, this threshold was 26,000 mf/mL (19,000-54,000) for females in the 31-40 age group; 24,000 mf/mL (18,000-41,000) for males in the 21-30 age group; 17,000 mf/mL (13,000-28,000) for males in the 31-40 age group; and 28,000 mf/mL (21,000-60,000) for males in the 41-50 age group.**”**

Please provide a little more background for why the >2.5% risk threshold of 30,000 is singled out in the text here and not 20,000. Since the LoaScope is calibrated to 20,000 and it corresponds to a >1% risk threshold, it feels a little odd not to also consider this threshold.

Response: We thank the reviewer for this pertinent remark. This was added in the results: “Considering the >1% predicted-risk (corresponding to the predicted overall risk when MFD exceeds 20,000 mf/mL) led to the same results, with threshold being of 18,000 mf/mL (13,000-42,000) for females in the 31-40 age group; 16,000 mf/mL (12,000-32,000) for males in the 21-30 age group; 12,000 mf/mL (9000-22,000) for males in the 31-40 age group; and 19,000 mf/mL (14,000-49,000) for males in the 41-50 age group.”

Line 306-307: I’m confused why this states that the risk is ~50,000 mf/ml when throughout the manuscript it refers to a risk of >1% occurring at 20,000 mf/ml. Please rephrase this sentence to clarify what you consider “risk” to be (e.g. is it >5% chance SAEs)?

Response: Indeed, this threshold was that retained for DEC. As the discussion has been revised to focus on IVM and to avoid diluting the message presenting to many results and pathological findings on DEC-related side effect, this sentence has been deleted, among others.

I note that the conclusion of the manuscript is to lower the threshold to 20,000 mf/mL; however, there is no mention of the fact that this recommendation aligns with the current threshold used for Test and Not Treat, which is the primary strategy by which individuals will be treated with IVM in Africa.

Response: We thank the reviewer for this valuable insight. This has been added to the manuscript “, aligning with the threshold retained in the Test and Not Treat strategy relying on the LoaScope in endemic areas”. Further aligning our recommendation with that applied in the Test and Not Streat strategy, corresponding to a 1% global risk according to our estimates, we refined our therapeutic propositions according to age and sex to match it.

“Aligning with the threshold retained in the Test and Not Treat strategy relying on the LoaScope in endemic areas that allows exclusion of individuals with an estimated MFD≥20,000 mf/mL, corresponding to a global risk of 1% in our estimations, and to match with the categories that are routinely used, we would advise that the threshold for IVM use in *L. loa* microfilaremic patients is lowered down to 8000 mf/mL for males between 20 and 50 years, or females in the 30-40 age group. To simplify therapeutic recommendations, the threshold of 8000 mf/mL could thus be recommended for all patients. Individuals harboring MFD above those thresholds should perhaps receive ALB or be considered for apheresis. New treatment options are currently being studied that could also constitute a valuable alternative, such as levamisole [70,71]. Given the lack of hindsight on safety of ALB at higher dosages in loiasis subjects (400 mg b.i.d. for 28 days, as used in some centers), and the recent concerns raised by two cases of SAEs in patients treated with ALB [31,32], it seems wise to keep with the protocol used by Klion *et al*, i.e. 200 mg b.i.d. during 21 days, and repeat if necessary [28]. Such a level of caution can be acceptable given the rarity of the disease outside endemic areas and the balance between benefits and risks.”

Reviewer #3: (No Response)
